# Preliminary Exploration of Potential Active Ingredients and Molecular Mechanisms of Yanggan Yishui Granules for Treating Hypertensive Nephropathy Using UPLC-Q-TOF/MS Coupled with Network Pharmacology and Molecular Docking Strategy

**DOI:** 10.1155/2024/7967999

**Published:** 2024-05-10

**Authors:** Fan Yang, Kailun Zhang, Xiaohua Dai, Weimin Jiang

**Affiliations:** ^1^Department of Cardiology, Affiliated Hospital of Nanjing University of Chinese Medicine, Nanjing, Jiangsu 210023, China; ^2^Department of Cardiology, The First Affiliated Hospital, Anhui University of Chinese Medicine, Hefei, Anhui 230000, China; ^3^College of Pharmacy, Anhui University of Chinese Medicine, Hefei, Anhui 230000, China

## Abstract

Hypertensive nephropathy (HN) is a prevalent complication of hypertension and stands as the second primary reason for end-stage renal disease. Research in clinical settings has revealed that Yanggan Yishui Granule (YGYSG) has significant therapeutic effects on HN. However, the material basis and action mechanisms of YGYSG against HN remain unclear. Consequently, this study utilized a comprehensive method integrating ultraperformance liquid chromatography-quadrupole time-of-flight mass spectrometry (UPLC-Q-TOF/MS), network pharmacology, and molecular docking to delineate the active ingredients and potential therapeutic mechanisms of YGYSG for treating HN. Firstly, sixty distinct components were recognized in total as potential active ingredients in YGYSG by UPLC-Q-TOF/MS. Subsequently, the mechanisms of YGYSG against HN were revealed for the first time using network pharmacology. 23 ingredients played key roles in the complete network and were the key active ingredients, which could affect the renin-angiotensin system, fluid shear stress and atherosclerosis, HIF-1 signaling pathway, and AGE-RAGE signaling pathway in diabetic complications by regulating 29 key targets such as TNF, IL6, ALB, EGFR, ACE, and MMP2. YGYSG could treat HN through the suppression of inflammatory response and oxidative stress, attenuating the proliferation of renal vascular smooth muscle cells, lessening glomerular capillary systolic pressure, and ameliorating renal dysfunction and vascular damage through the aforementioned targets and pathways. Molecular docking results revealed that most key active ingredients exhibited a high affinity for binding to the key targets. This study pioneers in clarifying the bioactive compounds and molecular mechanisms of YGYSG against HN and offers scientific reference into the clinical application.

## 1. Introduction

Hypertension is the most prevalent cardiovascular disease and a primary cause of premature death worldwide [1]. This condition is linked to organ damage, particularly in the kidneys [2]. With progression, hypertension can lead to hypertensive nephropathy (HN), characterized by a spectrum of pathological alterations such as vascular remodeling, interstitial fibrosis, glomerular damage, fibrinoid necrosis, atherosclerosis, oxidative stress, and inflammation [3, 4]. According to existing research, an appropriate control of high blood pressure is a key strategy for treating HN [5]. The five principal classes of antihypertensive drugs, including calcium antagonists, *β*-blockers, angiotensin-converting enzyme (ACE) inhibitors, thiazide diuretics, and angiotensin receptor blockers, are widely recognized for their efficacy in long-term management of hypertension [6]. However, these medications are not without side effects, which may include hypokalaemia, bronchial asthma, and lower limb edema [7, 8]. Considering the swift global rise in HN cases and the constraints of current treatments, it is critically important to identify new medications that offer effective therapeutic outcomes and minimal side effects.

It is common knowledge that prescriptions of traditional Chinese medicine (TCM) have pharmacological properties due to their multicomponent, multitarget, and multipathway and low side effects [9]. Yanggan Yishui Granule (YGYSG) was developed by the famous expert in TCM Professor Yixuan Zhou from the First Hospital Affiliated to Anhui University of Traditional Chinese Medicine after gaining extensive clinical experience and knowledge of herbal properties. YGYSG has been clinically applied to the HN for decades with remarkable curative effects, and previous studies of our research group have shown that it has the effects of improving proteinuria and protecting kidneys [10–12]. The formula includes Astragali Radix (ASR), Achyranthis Bidentatae Radix (ABR), Salviae Miltiorrhizae Radix et Rhizoma (SMR), Lycii Fructus (LYF), Euryales Semen (EUS), and Cuscutae Semen (CUS). The detailed information is presented in Table 1. According to the ancient TCM theory, LYF is the monarch drug in YGYSG, which has the effect of nourishing liver and kidney. CUS, ASR, and EUS are the minister drugs in YGYSG. Thereinto, CUS has the effects of consolidating the essence and tonifying the liver and kidney; ASR has the effects of invigorating “qi,” promoting “yang,” and solidifying the surface; EUS has the effects of strengthening the kidney and stopping spermatorrhea. SMR is the assistant drug and has the effects of promoting blood circulation and eliminating blood stasis. ABR is also an assistant drug that guides blood to the lower parts of the body and enhances the nourishing liver and kidney functions of LYF. Nonetheless, the active constituents of YGYSG and the underlying mechanisms of its therapeutic effect on HN are yet to be completely elucidated.

Ultraperformance liquid chromatography-quadrupole time-of-flight mass spectrometry (UPLC-Q-TOF/MS) has become increasingly useful due to its advantages of sensitivity, high resolution, and throughput. These attributes facilitate the swift identification and characterization of target components, effectively addressing issues such as component complexity and quantification challenges inherent in TCM analysis [13]. Network pharmacology is a promising approach in bioinformatics, systems biology, and polypharmacology owing to its cost-effectiveness in drug discovery. Its holistic theory closely aligns with that of the TCM theory [14]. The research process of network pharmacology mainly includes the identification of potential active ingredients, prediction of targets for drugs and specific diseases, and the construction and analysis of TCM prescriptions-targets-diseases network [15]. A large number of authoritative databases, such as TCMSP (Traditional Chinese Medicine System Pharmacology, https://old.tcmsp-e.com/tcmsp.php) [16], TCMID (Traditional Chinese Medicines Integrated Database, https://47.100.169.139/tcmid/) [17], BATMAN-TCM (Bioinformatics Analysis Tool for Molecular mechANism of TCM, https://bionet.ncpsb.org/batman-tcm/) [18], and HERB (a high-throughput experiment-and reference-guided database of TCM, https://herb.ac.cn/) [19], are used to collect potential active ingredients in TCM prescriptions. Databases used for drug-target prediction mainly include SwissTargetPrediction (https://swisstargetprediction.ch/) [20], SEA (similarity ensemble approach, https://sea.bkslab.org/) [21], and PharmMapper (https://www.lilab-ecust.cn/pharmmapper/index.html) [22]. Databases used for disease-target prediction mainly include DisGeNET (https://www.disgenet.org/home/) [23], OMIM (Online Mendelian Inheritance in Man, https://www.ncbi.nlm.nih.gov/omim) [24], and Drugbank (https://go.drugbank.com/) [25]. Cytoscape, a drug-target interaction prediction software [26], is usually used combined with the STRING database (Search Tool for the Retrieval of Interacting Genes/Proteins, https://cn.string-db.org/) [27] to screen key active ingredients and key targets. DAVID database (Database for Annotation, Visualization, and Integrated Discovery, https://David.ncifcrf.gov/) [28] is applied to further screen key signaling pathways. Then, the network of TCM prescriptions-key active ingredients-key targets-key signaling pathways-diseases is constructed by Cytoscape to preliminarily elucidate the molecular mechanisms of TCM prescriptions for treating diseases. Molecular docking is capable of assessing the dependability of outcomes predicted by network pharmacology through analyzing the binding modes and binding forces between drugs and targets [29]. This fusion of network pharmacology and molecular docking technology has gained widespread application in elucidating molecular mechanisms in TCM formulas in recent years. Ming's team delved into the mechanisms of the Longchai Jiangxue formula involved in treating polycythemia vera by using UPLC/Q-TOF-MS/MS alongside network pharmacology and molecular docking [30]. Similarly, Duan's group investigated the antibacterial properties and active components of tea-seed oil through the combined application of UPLC-Q-TOF/MS, network analysis, and molecular docking [31].

**Figure 1:**
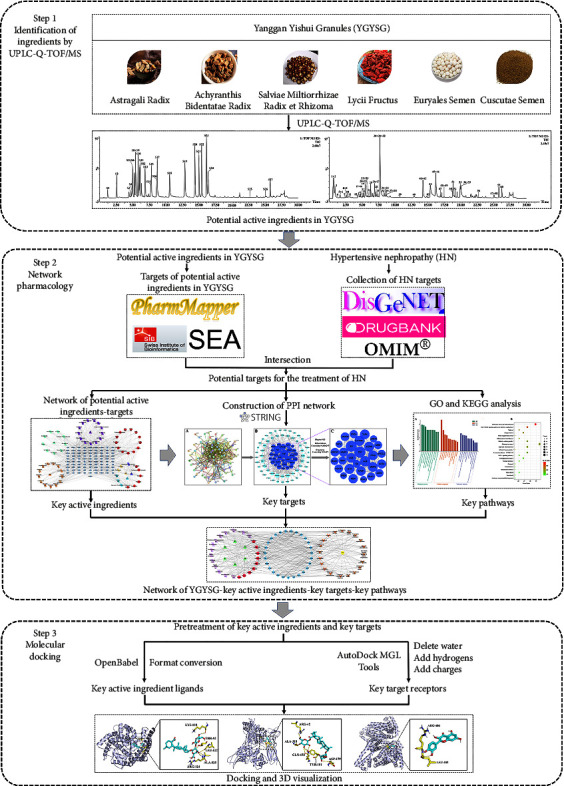
Flowchart of this current study.

In this regard, the present study employed an integrated and comprehensive strategy that combined UPLC-Q-TOF/MS coupled with network pharmacology and molecular docking to investigate the potential active ingredients and mechanisms underlying the curative effects of YGYSG in the treatment of HN. An optimized UPLC-Q-TOF/MS method was initially utilized to scrutinize the active components within YGYSG. Next, the network of YGYSG-key active ingredients-key targets-key pathways was constructed to uncover the potential mechanisms of YGYSG for treating HN using network pharmacology technology. Molecular docking was then utilized to substantiate the network pharmacology findings. This study lays a foundational theory for the development and clinical application of YGYSG.  Figure 1 displays a flowchart representing this study.

## 2. Materials and Methods

### 2.1. Chemicals and Materials

**Figure 2:**
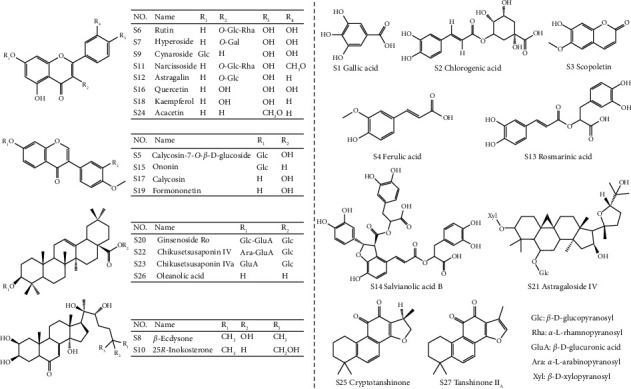
Chemical structures of the 27 reference standards used in this study.

The production of YGYSG was undertaken in The First Clinical Medical College at Anhui University of Chinese Medicine. A Milli-Q water purification system (Millipore, Bedford, MA, USA) was employed to acquire deionized water. For mass spectrometry analysis, formic acid of LC-MS grade was purchased from Sigma-Aldrich (St. Louis, MO, USA). Acetonitrile and methanol were LC-MS grade and obtained from Merck (Darmstadt, Germany). Overall, 27 chemical standards, including chikusetsusaponin IVa, chikusetsusaponin IV, ginsenoside Ro, 25*R*-inokosterone, *β*-ecdysone, acacetin, cymaroside, quercetin, gallic acid, oleanolic acid, hyperoside, chlorogenic acid, astragalin, kaempferol, cryptotanshinone, tanshinone II_A_, rosmarinic acid, calycosin-7-*O*-*β*-D-glucoside, salvianolic acid B, astragaloside IV, formononetin-7-*O*-*β*-D-glycoside, calycosin, formononetin, scopoletin, ferulic acid, rutin, and narcissoside, were purchased from Shanghai Yuanye Bio-Technology Co., Ltd. (Shanghai, China), and their purity levels exceeded 98% as determined through high-performance liquid chromatography (HPLC) analysis.  Figure 2 displays the chemical configurations of each reference standard.

### 2.2. Preparation of YGYSG Sample Solutions and Reference Compound Solutions

Regarding sample preparation, the dried herb samples were ground into uniform powders using a mill. The decoction piece powder of ASR (2 g), ABR (1 g), EUS (1 g), LYF (1 g), SMR (1 g), and CUS (1 g) were accurately weighed and mixed well. Precisely weighed herbal sample powder was dipped into a 200-mL glass-stoppered conical flask containing 140 mL 70% (v/v) methanol. The herbs were precisely and accurately weighed and subjected to ultrasonic extraction for 60 min at 25°C. During this process, to compensate for the loss of solvents, additional 70% (v/v) methanol was employed. After extraction, centrifugation of the extracts occurred at a speed of 13000 rpm for a duration of 15 minutes. The produced supernatants underwent filtration with a 0.22-*μ*m cellulose membrane filter prior to UPLC-Q-TOF/MS analysis.

For each of the 27 reference standards, stock solutions (around 2 mg/mL concentration) were concocted by dissolving close to 4 mg of every compound in 2 mL of 70% methanol. Subsequently, specific quantities of the aforementioned 27 reference compound stock solutions were combined to create the reference compound mixture working solution, resulting in an approximate concentration of 71.43 *μ*g/mL per reference compound. Prior to analysis, the reference compound mixture working solution was filtered through a 0.22-*μ*m cellulose membrane filter followed by storage at 4°C.

### 2.3. UPLC-Q-TOF-MS Analysis

Chromatographic analysis was conducted applying a Waters ACQUITY UPLC™ system (Waters, Milford, MA, USA) equipped with an autosampler and a binary solvent delivery manager. The Waters ACQUITY BEH C18 column (100 mm × 2.1 mm, 1.7 *μ*m) was applied with a column temperature of 30°C. The mobile phase system was composed of A (0.1% formic acid in water) and B (0.1% formic acid in acetonitrile). UPLC linear gradient elution conditions were 0–1 min, 5–10% B; 1–8 min, 10–27% B; 8–20 min, 27–35% B; 20–27 min, 35–90% B; 27–27.5 min, 90−5% B; and 27.5–30 min, 5% B. The injection volume of 2 *μ*L was utilized for reference compound mixture working solution and YGYSG sample solutions, with the flow rate being set at 0.4 mL/min.

A Waters SYNAPT G2-Si QTOF mass spectrometer fitted with an electrospray ionization (ESI) source (Waters MS Technologies, Manchester, UK) was used for the mass spectrometry analysis. Q-TOF-MS analysis was performed in the negative ion mode, and the parameters of mass spectrometry were as follows: source temperature, 120°C; capillary voltage, 2.5 kV; collision energy, 30–60 V; desolvation temperature, 450°C; cone voltage, 40 V; cone gas flow rate, 50 L/h; desolvation gas flow rate, 900 L/h; scan time, 0.2 s; and scan range, 50–1500 Da. The reference lock mass used was the [M − H]^−^ ion of leucine enkephalin (*m/z* 554.2615).

### 2.4. Target Acquisition of YGYSG and HN

The active constituents in YGYSG were characterized by UPLC-Q-TOF/MS to assist in network pharmacological studies. The SEA Search Server database (https://sea.bkslab.org/), Swiss target prediction database (https://swisstargetprediction.ch/), and PharmMapper database (https://www.lilab-ecust.cn/pharmmapper/) were used in the prediction of targets of the active ingredients. PubChem database (https://pubchem.ncbi.nlm.nih.gov/) provided the required compounds for the Swiss target prediction database and the SEA Search Server database in the SMILES format. Probable targets were identified according to the standard SMILES structure, limited to those annotated as “Homo sapiens,” and only the results with probability >0 were included. Disease-targets for “hypertensive nephropathy” were curated from the DrugBank database (https://go.drugbank.com/), OMIM database (https://www.omim.org/), and DisGeNET database (https://www.disgenet.org/), with human genes as a reference. All active ingredients-targets and disease-targets were standardized to gene symbols through the UniProt database (https://www.uniprot.org/), and duplicate values were removed. Finally, the common targets between the active components and diseases were considered potential targets of YGYSG for treating HN, as depicted in a Venn diagram.

### 2.5. Construction of Potential Active Ingredients-Targets Network and Screening of Key Active Ingredients

The common targets of components and disease, and potential active ingredients were introduced into Cytoscape 3.7.1, and the network of potential active ingredients-targets was constructed. The “Tools analysis network” feature in Cytoscape was used to screen the key active ingredients of YGYSG for HN based on the topological data, which include degree, closeness centrality (CC), and betweenness centrality (BC).

### 2.6. Construction of Protein-Protein Interaction Network and Screening of Key Targets

The common targets were imported into the STRING database (https://cn.string-db.org/) for the creation of a protein-protein interaction (PPI) network. Only protein interactions with a confidence level of >0.4 were considered. After sorting the protein interaction data, the information was prepared in a table format. Cytoscape 3.7.1 was used to analyze the common targets in the PPI network by applying three different analytical parameters such as degree, BC, and CC to discern key targets. Key targets, evaluated using various algorithms, were screened based on the parameter value greater than the median of three parameters, and the ultimate key targets were acquired through integration.

### 2.7. GO Function and KEGG Pathway Enrichment Analysis of Key Targets

For a comprehensive bioinformatics analysis, the key targets of YGYSG and HN were inputted into the DAVID database (https://David.ncifcrf.gov/) for annotation visualization and integrated discovery. Gene ontology (GO) enrichment and Kyoto Encyclopedia of Genes and Genomes (KEGG) pathway annotations of the key targets of YGYSG for treating HN were performed. The terms “Gene official symbol” and species “Homo sapiens” were chosen, and a *P* value under 0.05 was considered a marker of statistical importance. In addition, the top 20 signaling pathways were considered key pathways.

### 2.8. Construction of YGYSG-Key Active Ingredients-Key Targets-Key Pathways Network

To analyze the association among HN, the key active ingredients of YGYSG, the key targets, and the key signaling pathways, a network of YGYSG-key active ingredients-key targets-key pathways was constructed by Cytoscape 3.7.1.

### 2.9. Molecular Docking

The dependability of the outcome from network pharmacology was further verified through molecular docking techniques. The three-dimensional configurations of the top 10 key active ingredients in YGYSG were obtained from the PubChem database (https://pubchem.ncbi.nlm.nih.gov/). Subsequently, the molecular structures of the top 10 key targets from the PPI network were retrieved from the RCSB PDB database (https://www.rcsb.org/). The protein receptors were then imported into AutoDockTools version 1.5.7 by adding hydrogen atoms, assigning charges, and removing water molecules. The AutoDock Vina software program was utilized to perform molecular docking, wherein the affinity of the ligands and protein receptors, the stability of the ligand-receptor binding, and the energy value of the binding process were evaluated. Visualization of the molecular mocking outcomes was achieved using PyMOL software. A docking was considered successful and valid if the binding free energy was at or below −5.0 kcal/mol.

## 3. Results

### 3.1. Identification of Chemical Constituents in YGYSG by UPLC-Q-TOF/MS

**Figure 3:**
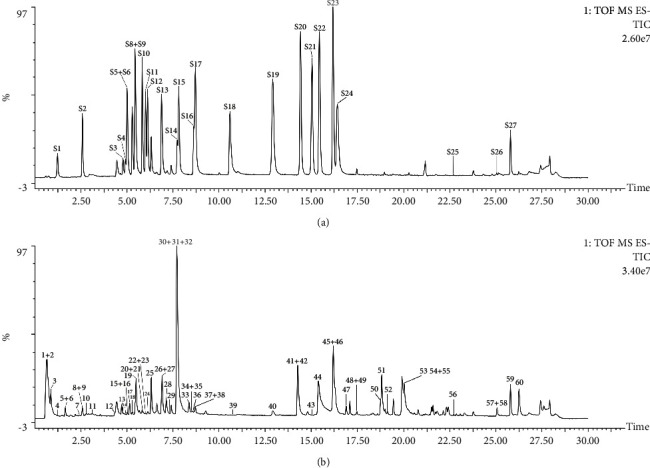
Representative total ion current chromatograms of the reference standards (a) and the samples of YGYSG (b) in the negative ion mode. The sequence of the reference standards (S1–S27) is consistent with Figure 2. The sequence of the identified ingredients (1–60) is in conformity with Table 2.

UPLC-Q-TOF/MS analysis was employed to delineate the chemical constituents of YGYSG. The total ion current (TIC) plots of YGYSG in negative ion modes are exhibited in Figure 3. Out of 60 identified compounds (identified as 1–60), 27 were clearly determined by contrasting their retention times and precise masses against the reference standards. The remaining samples were tentatively assigned by aligning their empirical molecular formulas and characteristic fragment ions with those found in existing published compounds from similar studies [32–52]. The chromatographic behaviors of certain chemical constituents served as additional information for pinpointing the isomers. Detailed structural identification of these components of YGYSG is presented in Table 2.

The results revealed that the 60 components included 19 phenolic acids (**4–12**, **14**, **22**, **26–31**, **33-34**), 15 saponins (**41–55**), 13 flavonoids (**15–18**, **20**, **23–25**, **32**, **36**, **38–40**), 3 fatty acids (**3**, **58**, **60**), 2 sterones (**19**, **21**), 2 quinones (**56**, **59**), and 6 other compounds (**1-2**, **13**, **35**, **37**, **57**). Specifically, *β*-ecdysone (**19**), 25*R*-inokosterone (**21**), ginsenoside Ro (**42**), and zingibroside *R*_1_ (**54**) were considered the main bioactive constituents in ASR. Formononetin (**40**), astragaloside IV (**43**), soyasaponin I (**48**), and astragaloside I (**50**) were major active ingredients in ABR. Lithospermic acid (**30**), salvianolic acid B (**31**), cryptotanshinone (**56**), and tanshinone II_A_ (**59**) were main active components in SMR. Caffeic acid (**11**), ginsenoside Ro (**13**), ferulic acid (**14**), and narcissoside (**23**) were considered the main bioactive components in LYF. Protocatechualdehyde (**8**), astragalin (**24**), quercetin (**36**), and kaempferol (**39**) were major active ingredients in EUS. Lastly, chlorogenic acid (**9**), rutin (**15**), isoquercetin (**17**), and hyperoside (**18**) served as primary active compounds in CUS. These compounds, which have multiple pharmacological effects, may be the potential active ingredients of YGYSG against HN.

### 3.2. Acquisition of Potential Active Ingredients and HN Targets

**Figure 4:**
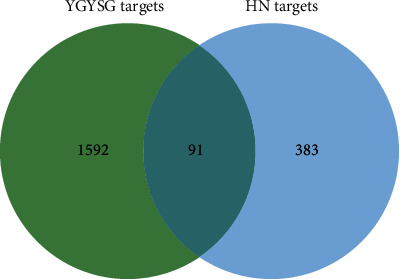
Venn diagram of the common targets of YGYSG and HN.

Upon identifying 60 compounds as potential active constituents, a comprehensive analysis yielded 1683 compound-related targets postduplication removal. Then, from the three specified online databases, a sum of 474 targets related to HN was amassed after removing duplication, which were further overlapped with the probable targets of the active ingredients in YGYSG. This resulted in the identification of 91 overlapping targets, posited as candidates for the treatment of HN. The intersection of YGYSG and HN targets is depicted in a Venn diagram (Figure 4).

### 3.3. Construction of Potential Active Ingredients-Targets Network and Screening of Key Active Ingredients

**Figure 5:**
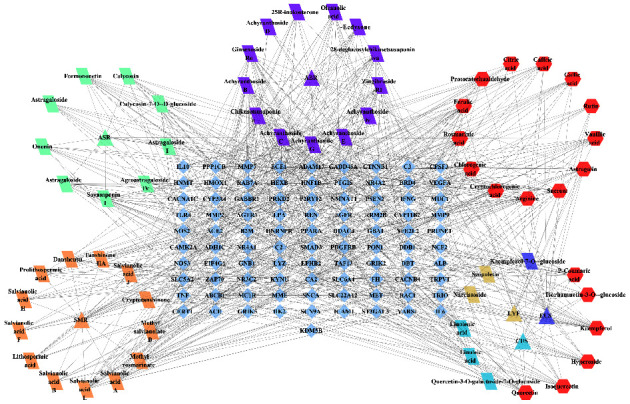
Network construction of the potential active ingredients-targets of YGYSG in the treatment of HN. Triangles represent the TCMs of YGYSG, parallelograms depict the exclusive active components of different TCMs, hexagons represent the common active compounds of these TCMs, diamonds symbolize the targets, and blue acronyms represent the gene name of the targets.

The network of potential active ingredients-targets (Figure 5) with 157 nodes and 803 edges was developed using Cytoscape 3.7.1. Network topology analysis revealed 23 components, including chikusetsusaponin IVa, ginsenoside Ro, soyasaponin I, and astragalin, which exhibited degree values, BC, and CC above the median (Table 3). These components are likely key active ingredients in YGYSG for treating HN and demonstrate the synergistic effect inherent in TCM.

### 3.4. Construction of PPI Network and Screening of Key Targets

**Figure 6:**
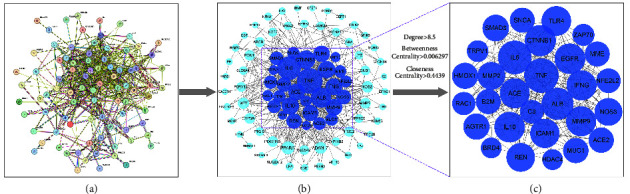
Key targets obtained from PPI network analysis. (a) Target interaction in the STRING database. (b) PPI network of YGYSG in the treatment of HN. (c) Key targets of YGYSG in the treatment of HN. Each node represents a target in the PPI network, and each edge indicates the interaction between adjacent nodes. The greater the number of adjacent nodes, the greater the probability of becoming a key target.

The 91 common targets of YGYSG and HN were integrated into the STRING database to generate a PPI network with 86 nodes and 543 edges (Figure 6(a)). Cytoscape 3.7.1 was employed to delve deeper into how targets interacted. As a target's degree increases, so does its node size and the intensity of its color (Figure 6(b)). With the help of a network analysis function, the target genes greater than the median of degree value (>8.5), BC (>0.006297), and CC (>0.4439) were considered key targets. 29 targets met these criteria, suggesting their potential role in treating HN (Figure 6(c)), which includes tumor necrosis factor (TNF), interleukin-6 (IL6), ACE, albumin (ALB), and epidermal growth factor receptor (EGFR), among others. Topological information of key targets in the YGYSG for treating HN is shown in Table 4.

### 3.5. GO Function and KEGG Enrichment Analyses of Key Targets

**Figure 7:**
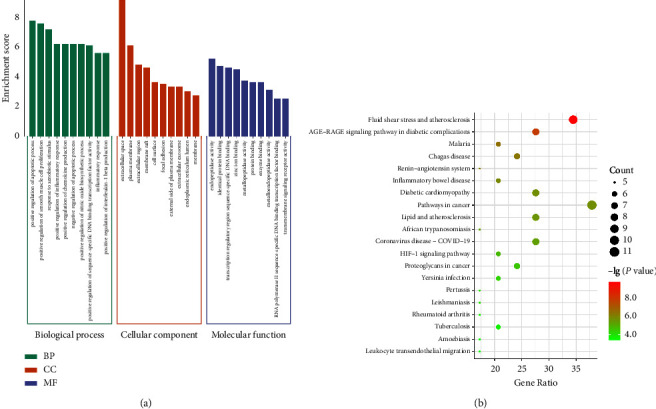
GO function analysis (a) and KEGG pathway analysis (b) of the key targets of the YGYSG in the treatment of HN.

Further analysis of the 29 key targets identified 279 GO functional enrichment analyses and 84 KEGG pathway enrichments. The gene ontology (GO) study covered 215 biological mechanisms, 31 cellular elements, and 33 molecular activities.  Figure 7(a) displays the leading 10 GO terms for every category. The top 10 GO terms for each category are presented in Figure 7(a). Notably, the leading ten biological processes were the positive regulation of the apoptotic process, positive regulation of smooth muscle cell proliferation, response to xenobiotic stimulus, and positive regulation of inflammatory response. The main cellular components included the extracellular space, plasma membrane, and extracellular region. The main molecular functions included transcription regulatory region sequence-specific DNA binding, identical protein binding, and endopeptidase activity. The main pathways involved were the AGE-RAGE signaling pathway in diabetic complications, HIF-1 signaling pathway, fluid shear stress and atherosclerosis, and renin-angiotensin system (Figure 7(b)).

### 3.6. Construction of YGYSG-Key Active Ingredients-Key Targets-Key Pathways Network

**Figure 8:**
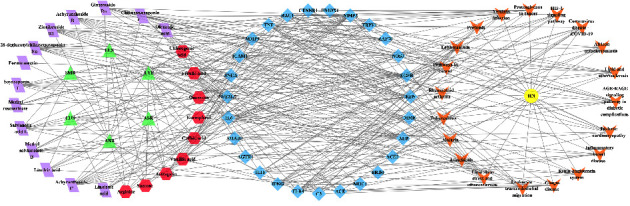
Network construction of the YGYSG-key active ingredients-key targets-key pathways of YGYSG against HN. Green triangles represent the TCMs of YGYSG, purple parallelograms symbolize the unique key active ingredients of various TCMs, red hexagons denote the common key active ingredients of these TCMs, blue diamonds represent the key targets, orange V shapes represent the key pathways, and yellow circles represent HN.

The KEGG pathway enrichment results enabled the construction of a network diagram of YGYSG-key active ingredients-key targets-key pathways (Figure 8), mapping the relationships between 29 protein targets, 23 potential bioactive compounds, and 20 signaling pathways. This network diagram serves as a foundational model for understanding the therapeutic mechanisms of YGYSG in the treatment of HN.

### 3.7. Molecular Docking

**Figure 9:**
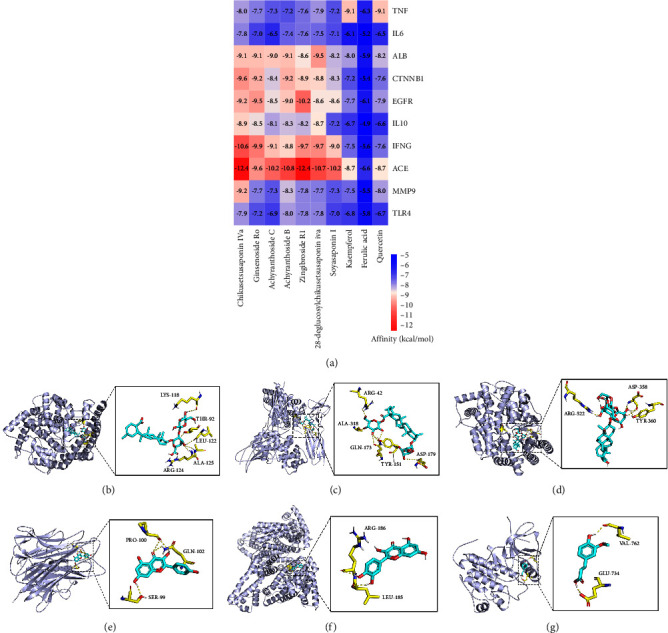
Molecular docking results of top 10 key active ingredients and key targets of YGYSG against HN. (a) Heatmap of the docking scores of top 10 key active ingredients and key targets of YGYSG. Molecular docking diagram of (b) zingibroside R1 with ACE, (c) chikusetsusaponin IVa with IFNG, (d) soyasaponin I with ACE, (e) kaempferol with TNF, (f) quercetin with ALB, and (g) ferulic acid with EGFR.

Figure 9(a) illustrates the docking specifics for each target along with the associated free energy expenses. Most of all possess great binding affinity, with binding energies of less than −7.0 kcal/mol. These results suggest that nearly all key active ingredients and targets have the ability to bind naturally, showing considerable affinity. The representative molecular docking interactions of six groups were visualized using PyMOL software (Figures 9(b)–9(g)), highlighting the predominant hydrogen bond interactions. For instance, the docking simulation of zingibroside R1 with ACE (Figure 9(b)) demonstrates the establishment of five hydrogen bonds between the ligand and residues of LYS-118, THR-92, LEU-122, ALA-125, and ARG-124.

## 4. Discussion

The current understanding of modern medicine identifies the activation of the renin-angiotensin system, vascular endothelial dysfunction, oxidative stress, and inflammation as central pathological processes in HN. It is a high-burden disorder and ranks as the second primary reason for terminal renal disease [53]. Given these circumstances, the discovery of safe and effective treatments for patients with HN is critical. YGYSG is beneficial for HN, as established by prior research. Employing a combination of UPLC-Q-TOF/MS, network pharmacology, and molecular docking validation, this study explored the potentially effective ingredients and mechanisms of YGYSG in the amelioration of HN. Initially, UPLC-Q-TOF/MS was used to profile the chemical constituents of YGYSG, identifying 60 components. The investigation revealed that YGYSG comprises 23 key active ingredients for treating HN, predominantly flavonoids, saponins, and phenolic acids. Further analysis involving the PPI network, KEGG pathway enrichment, and the network of YGYSG-key active ingredients-key targets-key pathways indicated that these active ingredients likely confer therapeutic effects through the modulation of 29 key targets and 20 key signaling pathways.

An increasing amount of evidence suggests a significant link between inflammation and hypertension. Chronic kidney disease features heightened levels of pro-inflammatory cytokines, including IL-1, IL-6, and TNF-*α* [54]. TNF-*α*, a pro-inflammatory cytokine, is a pathogenic factor implicated in renal injury by inducing apoptosis of renal epithelial cells and exacerbating inflammation, which further aggravates renal damage. Moreover, TNF-*α* has a direct cytotoxic influence on glomerular cells, mesangial cells, and renal epithelial cells [55]. IL-6, a multifunctional cytokine, plays a role in managing acute phase reactions, hematopoiesis, cellular regeneration, and immune responses [56]. The induction of IL-6 by TNF-*α* contributes to oxidative stress and inflammation and the subsequent deterioration of renal tissue [57]. Chikusetsusaponin IVa is an active ingredient in ABR and exerts anti-inflammatory properties by downregulating inflammatory factors such as TNF-*α* and IL-6 [58]. Ginsenoside Ro, an oleanolic saponin of ABR [59], has been demonstrated to inhibit the inflammatory response caused by TLR4 [60]. Chlorogenic acid, a phenolic acid derived from LYF, SMR, and CUS, significantly reduces the levels of TLR4, IL-1*β*, and TNF-*α* and renal inflammation [61]. Ferulic acid, a common plant-based phenolic acid, has renal-protective effects through its antioxidant, anti-inflammatory, lipid-lowering, antifibrotic, antiapoptotic, and autophagy-inducing properties [62]. Theoretical approaches such as molecular docking have confirmed that ferulic acid inhibited TNF-*α* and IL-6 at the structural level. Consequently, the presence of ferulic acid in YGYSG could potentially hinder the development and progression of HN by dampening the inflammatory response.

Oxidative stress is crucial in the pathology of vascular and renal inflammation, serving both as a catalyst and as a byproduct of inflammatory responses [63]. Vanillic acid is a phenolic acid identified from LYF, SMR, and ASR in this study and has the effect of improving systolic blood pressure [64]. Early study has shown that vanillic acid significantly inhibits the activity of oxidative stress markers in kidney tissue [65]. Ginsenoside Ro is a crucial ingredient in augmenting the protection of renal function within ABR, exhibiting pharmacological benefits through antiapoptotic and antioxidant activities [66]. NFE2L2 is a potential druggable target that can alleviate various redox-induced tissue damage by boosting its activity [67]. Formononetin, one of the main active ingredients in ASR, inhibits the excessive production of reactive oxygen species (ROS) and reduces oxidative damage by upregulating the expression of NFE2L2 [67]. Oxidative stress, alongside matrix metalloproteinase (MMP) activity, is present in the cardiovascular system during hypertension, contributing to cardiac myocyte hypertrophy and dysfunction. Quercetin, a flavonoid, suppresses the MMP-2 activity, thereby mitigating hypertrophic vascular remodeling associated with hypertension [68]. Additionally, the accumulation of AGE can accelerate during oxidative stress. The activation of RAGE by AGEs results in the creation of ROS and amplifies inflammation, contributing to the progressive alteration in renal structure and loss of renal function [69]. AGEs also bind to RAGE, activating the HIF-1 signaling pathway [70]. HIF-1, a central regulator of cellular response to hypoxia, is implicated in various kidney pathologies, including renal fibrosis [71]. HIF-1 signaling pathway is linked with the progression of chronic kidney injury. It has been proposed that kidney injury may be improved by inhibiting the HIF-1 signaling pathway [72, 73].

Activation of the renin-angiotensin system is recognized as a significant pathological factor contributing to HN. Inhibiting this system has been effective in mitigating renal dysfunction and vascular damage [74]. ACE, crucial in the renin-angiotensin system [75], manages blood pressure through the transformation of angiotensin I into angiotensin II (Ang II). Suppressing the ACE activity results in a reduction of Ang II production, thereby causing vasodilation and lowered blood pressure, and the antihypertensive effect of ASR was realized in this way [76]. Moreover, Ang II directly impacts the cells of renal vascular smooth muscles, wherein it induces vasoconstriction of both efferent and afferent renal arterioles, causing both increased glomerular pressure and decreased renal blood flow. This also increases the sensitivity of the tubuloglomerular feedback within the kidneys [5]. Soyasaponin I, a bioactive compound present in ASR, belongs to the triterpene saponins. Earlier research has verified that soyasaponin I was a natural inhibitor of renin and showed inhibitory effects on the renin-angiotensin system [77]. Caffeic acid, with its diverse pharmacological effects, which include antioxidant, anti-inflammatory, and antihypertensive activities [78], notably inhibits ACE, further influencing the renin-angiotensin system. Caffeic acid is notable for its capacity to diminish the ACE activity, a crucial bioactive compound in the renin-angiotensin system. Furthermore, studies have shown that caffeic acid can lower systolic blood pressure and attenuate the proliferation of vascular smooth muscle cells [79].

Simultaneous chronic hypertension may lead to the thickening of the intima and narrowing of the lumen in renal arteries and arterioles, as well as tubular atrophy, interstitial fibrosis, and glomerulosclerosis, which usually indicate the occurrence of hypertensive nephropathy [80]. Consequently, HN may progress into a distinct clinical entity known as atherosclerotic nephropathy. Fluid shear stress and atherosclerosis is the crucial pathway of inhibiting inflammation, vasodilation, and anti-atherosclerosis [81]. Shear stress from regions of disease causes the expression and deposition of fibronectin via the inflammatory transcription factor NF-*κ*B activation, thus establishing a pro-inflammatory positive feedback loop that sustains inflammation in atherosclerotic lesions [82]. Therapeutically, disrupting this loop could prove beneficial in treating HN by decelerating the progression of atherosclerosis.

EGFR activation is linked to damage and loss of podocytes, specialized cells critical for kidney function, while the deletion of EGFR in podocytes mitigates glomerular injury and offers protection against kidney damage in the context of diabetic nephropathy [83]. ALB ranks as the predominant protein found in human blood. Notably, urinary ALB has been identified earlier as a contributing factor to the development of hypertensive nephrosclerosis [84]. Lower ALB levels are associated with decreased activity of transforming growth factor-*β*1, alongside diminished pathology in tubulointerstitial, glomerular, and podocyte [85]. Molecular docking studies have shown that EGFR and ALB exhibit the strongest binding affinity to the compounds zingibroside R1 and 28-deglucosylchikusetsusaponin IVa, respectively. These findings suggest that these compounds in the YGYSG could have therapeutic effects in HN by modulating the levels of EGFR and ALB. In summary, YGYSG offers a significant therapeutic potential for HN by leveraging a strategy of multicomponent and multitarget.

## 5. Conclusions

As far as we are aware, this is the first study to reveal the bioactive compounds and molecular mechanisms of YGYSG in the treatment of HN based on a comprehensive research strategy of UPLC-Q-TOF/MS coupled with network pharmacology and molecular docking. Through this approach, 60 potential active ingredients within YGYSG, including 19 phenolic acids, 15 saponins, 13 flavonoids, 3 fatty acids, 2 sterones, 2 quinones, and 6 other compounds, were detected for the first time using UPLC-Q-TOF/MS. Further analysis deemed 23 of these constituents as key active ingredients of YGYSG against HN. These key active ingredients were suggested to mitigate inflammation and oxidative stress, curtail the proliferation of renal vascular smooth muscle cells, decrease the glomerular capillary systolic pressure, and improve the renal dysfunction and vascular injury by acting on the 29 key targets including TNF, IL6, ALB, EGFR, ACE, and MMP2, involving the fluid shear stress and atherosclerosis, HIF-1 signaling pathway, renin-angiotensin system, and AGE-RAGE signaling pathway in diabetic complications. Molecular docking has confirmed a strong affinity between nearly all key active ingredients and key targets. This study lays the groundwork for the future exploration and development of YGYSG as a treatment for HN, marking a pioneering step in understanding its active components and mechanisms.

## Figures and Tables

**Table 1:** Information on the composition of YGYSG.

Pharmaceutical name	Chinese name	Weight (g)	Proportion (%)	Abbreviation
Astragali Radix	Huangqi	500	28.57	ASR
Achyranthis Bidentatae Radix	Niuxi	250	14.29	ABR
Salviae Miltiorrhizae Radix et Rhizoma	Danshen	250	14.29	SMR
Lycii Fructus	Gouqi	250	14.29	LYF
Euryales Semen	Qianshi	250	14.29	EUS
Cuscutae Semen	Tusizi	250	14.29	CUS

**Table 2:** Identification of chemical constituents in YGYSG based on UPLC-Q-TOF/MS.

No	Identification	Molecular formula	*t*_*R*_ (min)	[M − H]^−^ (mass accuracy, ppm)	[M − H + HCOOH]^−^ (mass accuracy, ppm)	Fragment ions of [M − H]^−^ at low energy (mass accuracy <5 ppm)	Source	Ref
1	Arginine	C_6_H_14_N_4_O_2_	0.68	173.1050 (6.4)	—	149.0476; 114.0183	ABR, ASR, EUS	[34, 35]
2	Sucrose	C_12_H_22_O_11_	0.68	341.1065 (−5.6)	387.1132 (−1.8)	191.0565; 179.0534	ABR, LYF, ASR, EUS	[34, 35]
3	Citric acid	C_6_H_8_O_7_	0.89	191.0188 (−2.1)	—	111.0076	SMR, EUS	[35, 36]
4^*∗*^	Gallic acid	C_7_H_6_O_5_	1.24	169.0149 (7.1)	—	125.0232	SMR, LYF, EUS	[35, 37]
5	Vanillic acid	C_8_H_8_O_4_	1.67	167.0337 (−4.2)	—	123.0450; 108.0206	SMR, LYF, ASR	[36, 38]
6	Danshensu	C_9_H_10_O_5_	1.67	197.0444 (−3.0)	—	179.0351; 151.0396; 135.0429	SMR	[36]
7	Quercetin-3-*O*-galactoside-7-*O*-glucoside	C_27_H_30_O_17_	2.43	625.1395 (−1.6)	—	463.0848; 301.0323	CUS	[33]
8	Protocatechualdehyde	C_7_H_6_O_3_	2.60	137.0245 (4.4)	—	108.0229	SMR, EUS	[36, 39]
9^*∗*^	Chlorogenic acid	C_16_H_18_O_9_	2.60	353.0894 (5.9)	—	191.0565; 161.0246; 135.0429	SMR, LYF, EUS	[40]
10	Cryptochlorogenic acid	C_16_H_18_O_9_	2.80	353.0851 (−6.2)	—	191.0565; 135.0429	SMR, EUS	[40, 41]
11	Caffeic acid	C_9_H_8_O_4_	3.14	179.0351 (3.9)	—	135.0429; 117.0349	SMR, LYF, EUS, CUS	[36]
12	*P*-coumaric acid	C_9_H_8_O_3_	4.35	163.0396 (0.6)	—	146.9641; 119.0489	LYF, CUS	[40]
13^*∗*^	Scopoletin	C_10_H_8_O_4_	4.79	191.0345 (0.5)	—	176.0138; 148.0165	LYF	[42]
14^*∗*^	Ferulic acid	C_10_H_10_O_4_	4.92	193.0517 (8.3)	—	179.0351; 153.9211; 134.0352	SMR, LYF	[36, 39]
15^*∗*^	Rutin	C_27_H_30_O_16_	5.01	609.1442 (−2.3)	—	301.0323; 283.0579; 255.0281	LYF, EUS, CUS	[35, 40]
16^*∗*^	Calycosin-7-*O*-*β*-D-glucoside	C_22_H_22_O_10_	5.02	445.1141 (1.3)	491.1208 (3.7)	283.0579; 268.0368	ASR	[51]
17	Isoquercetin	C_21_H_20_O_12_	5.16	463.0897 (4.3)	—	301.0323	LYF, EUS, CUS	[35, 40]
18^*∗*^	Hyperoside	C_21_H_20_O_12_	5.29	463.0897 (4.3)	—	300.0269; 271.0240; 255.0281	LYF, EUS, CUS	[35, 40]
19^*∗*^	*β*-ecdysone	C_27_H_44_O_7_	5.50	479.3025 (3.3)	525.3052 (−2.3)	443.2782; 319.1902; 301.1744	ABR	[43]
20	Kaempferol-7-*O*-*β*-glucoside	C_21_H_20_O_11_	5.84	447.0933 (1.3)	—	285.0397	EUS	[32]
21^*∗*^	25*R*-inokosterone	C_27_H_44_O_7_	5.84	479.3025 (3.3)	525.3052 (−2.3)	443.2782; 319.1902; 301.1744	ABR	[43]
22	Salvianolic acid J	C_27_H_22_O_12_	6.01	537.1049 (3.0)	—	295.0601; 185.0217; 109.0275	SMR	[36]
23^*∗*^	Narcissoside	C_28_H_32_O_16_	6.01	623.1621 (1.4)	669.1641 (−3.9)	315.0549	LYF	[38]
24^*∗*^	Astragalin	C_21_H_20_O_11_	6.12	447.0933 (1.3)	—	284.0307; 191.0565; 179.0351	ABR, LYF, ASR, EUS, CUS	[35, 40]
25	Isorhamnetin-3-*O*-*β-*glucoside	C_22_H_22_O_12_	6.33	477.1041 (1.7)	—	314.0424; 285.0397; 243.0303	EUS, CUS	[40]
26^*∗*^	Rosmarinic acid	C_18_H_16_O_8_	6.89	359.0764 (−0.8)	—	197.0444; 179.0321	SMR, LYF	[36, 39]
27	Prolithospermic acid	C_18_H_14_O_8_	6.89	357.0623 (3.6)	—	135.0429; 109.0299	SMR	[41]
28	Salvianolic acid H	C_27_H_22_O_12_	7.14	537.0995 (−7.1)	—	295.0601; 185.0217; 109.0275	SMR	[36]
29	Salvianolic acid F	C_17_H_14_O_6_	7.33	313.0798 (8.6)	—	269.0916; 237.0610	SMR	[39]
30	Lithospermic acid	C_27_H_22_O_12_	7.72	537.0995 (−7.1)	—	295.0601; 185.0217; 109.0275	SMR	[36]
31^*∗*^	Salvianolic acid B	C_36_H_30_O_16_	7.72	717.1456 (0.0)	—	519.0942; 321.0376; 295.0601	SMR	[36]
32^*∗*^	Ononin	C_22_H_22_O_9_	7.72	429.1183 (−0.7)	475.1240 (0.0)	267.0658	ASR	[50]
33	Salvianolic acid L	C_36_H_30_O_16_	8.38	717.1456 (0.0)	—	519.0942; 321.0376; 295.0601	SMR	[41]
34	Salvianolic acid A	C_26_H_22_O_10_	8.52	493.1138 (0.6)	—	295.0601; 179.0351	SMR	[37]
35	Methyl rosmarinate	C_19_H_18_O_8_	8.52	373.0927 (1.1)	—	193.0486; 135.0429	SMR	[37]
36^*∗*^	Quercetin	C_15_H_10_O_7_	8.63	301.0323 (−8.3)	—	178.9986; 151.0033	LYF, EUS, CUS	[35]
37	Methyl salvianolate B	C_37_H_32_O_16_	8.72	731.1608 (−0.5)	—	533.1063; 353.0637; 335.0533	SMR	[39]
38^*∗*^	Calycosin	C_16_H_12_O_5_	8.72	283.0617 (3.9)	—	268.0368; 265.0476	ASR	[44]
39^*∗*^	Kaempferol	C_15_H_10_O_6_	10.60	285.0397 (−0.7)	—	255.0281; 151.0033	LYF, EUS, CUS	[35, 40]
40^*∗*^	Formononetin	C_16_H_12_O_4_	12.91	267.0658 (0.4)	—	252.0439	ASR	[50]
41	Achyranthoside D	C_53_H_82_O_25_	14.26	1117.5093 (2.3)		1041.4930; 997.5016	ABR	[45]
42^*∗*^	Ginsenoside Ro	C_48_H_76_O_19_	14.26	955.4927 (2.5)	1001.4966 (0.9)	793.4353; 731.4376	ABR	[45]
43^*∗*^	Astragaloside IV	C_41_H_68_O_14_	15.04	783.4534 (0.4)	829.4622 (4.3)	471.3538	ASR	[44]
44	Achyranthoside B	C_47_H_70_O_20_	15.38	953.4409 (2.8)	—	793.4353; 631.3819	ABR	[47]
45^*∗*^	Chikusetsusaponin IVa	C_42_H_66_O_14_	16.17	793.4353 (−2.6)	839.4421 (−1.0)	631.3819; 613.3784; 587.3987; 569.3868	ABR	[46]
46	Achyranthoside C	C_47_H_72_O_20_	16.19	955.4576 (3.9)		835.4469; 793.4353; 631.3876; 569.3868	ABR	[45]
47	Astragaloside II	C_43_H_70_O_15_	16.89	825.4647 (1.3)	871.4713 (2.5)	807.4373; 489.3029	ASR	[48]
48	Soyasaponin I	C_48_H_78_O_18_	17.47	941.5114 (0.4)	—	633.3952; 457.3712	ASR	[50]
49	Agroastragaloside IV	C_49_H_80_O_20_	17.47	987.5132 (−3.3)	—	455.3499	ASR	[51]
50	Astragaloside I	C_45_H_72_O_16_	18.76	867.4742 (0.0)	913.4799 (0.2)	573.2902; 489.3582	ASR	[48]
51	Achyranthoside G	C_47_H_72_O_20_	18.82	955.4576 (3.9)	—	835.4535; 793.4417; 631.3819; 569.3868	ABR	[47]
52	Achyranthoside E	C_46_H_70_O_19_	19.12	925.4456 (2.5)	—	793.4353; 569.3814; 455.3548	ABR	[47]
53	Achyranthoside iv	C_41_H_60_O_15_	19.92	791.3865 (1.4)	—	631.3876; 455.3548	ABR	[52]
54	Zingibroside *R*_1_	C_42_H_66_O_14_	20.04	793.4353 (−2.1)		731.4376; 631.3762; 613.3728	ABR	[45]
55	28-deglucosylchikusetsusaponin IVa	C_36_H_56_O_9_	20.04	631.3876 (4.8)	677.3908 (1.0)	555.3690; 509.3671	ABR	[45]
56^*∗*^	Cryptotanshinone	C_19_H_20_O_3_	22.70	295.1343 (3.0)	—	174.9533; 146.9641	SMR	[36]
57^*∗*^	Oleanolic acid	C_30_H_48_O_3_	25.07	455.3548 (5.1)	—	407.1727; 391.2007	ABR	[49]
58	Linolenic acid	C_18_H_30_O_2_	25.07	277.2184 (5.8)	—	146.9641	CUS	[40]
59^*∗*^	Tanshinone II_A_	C_19_H_18_O_3_	25.80	293.1177 (−0.3)		163.1122	SMR	[39]
60	Linoleic acid	C_18_H_32_O_2_	26.26	279.2328 (1.4)	—	146.9641; 116.9267	CUS	[40]

^*∗*^The chemical constituents unambiguously identified with reference standards comparison.

**Table 3:** Topological information of key active ingredients in the YGYSG for treating HN.

Ingredient name	Degree	Betweenness centrality	Closeness centrality	PubChem CID	Source
Chikusetsusaponin IVa	24	0.02606093	0.42975207	13909684	ABR
Ginsenoside Ro	24	0.02382803	0.42975207	11815492	ABR
Achyranthoside C	24	0.02307622	0.42975207	78172884	ABR
Achyranthoside B	23	0.02190101	0.42739726	134692504	ABR
Zingibroside *R*_1_	23	0.02159459	0.42739726	10395524	ABR
28-deglucosylchikusetsusaponin IVa	23	0.01989671	0.42739726	176079	ABR
Soyasaponin I	22	0.02142234	0.42276423	122097	ASR
Kaempferol	21	0.03058414	0.42048518	5280863	LYF, EUS, CUS
Ferulic acid	20	0.04296974	0.42048518	445858	SMR, LYF
Quercetin	19	0.02512998	0.416	5280343	LYF, EUS, CUS
Arginine	19	0.05844239	0.41823056	6322	ABR, ASR, EUS
Salvianolic acid L	19	0.02023113	0.4137931	11765414	SMR
Astragalin	17	0.01878109	0.416	5282102	ABR, LYF, EUS, ASR, CUS
Methyl salvianolate B	17	0.04482492	0.4116095	46917416	SMR
Oleanolic acid	17	0.04339334	0.39897698	10494	ABR
Chlorogenic acid	16	0.02721358	0.4116095	1794427	SMR, LYF, CUS
Sucrose	16	0.0200863	0.4073107	5988	ABR, LYF, ASR, EUS
Linoleic acid	16	0.02580897	0.39897698	5280450	CUS
Vanillic acid	15	0.03647452	0.40944882	8468	SMR, LYF, ASR
Methyl rosmarinate	15	0.01749654	0.40944882	6479915	SMR
Linolenic acid	15	0.02902451	0.39694656	5280934	CUS
Caffeic acid	14	0.02581528	0.4073107	689043	SMR, LYF, EUS, CUS
Formononetin	14	0.03260603	0.40519481	5280378	ASR

**Table 4:** Topological information of key targets in the YGYSG for treating HN.

Target name	UniProt ID	Degree	Betweenness centrality	Closeness centrality
TNF	P01375	47	0.12689366	0.65384615
IL6	P05231	44	0.07134401	0.62043796
ALB	P02768	39	0.05290924	0.59027778
CTNNB1	P35222	36	0.13803051	0.59440559
EGFR	P00533	34	0.05880376	0.57823129
IL10	P22301	34	0.02450863	0.57432432
IFNG	P01579	32	0.07380561	0.56291391
ACE	P12821	32	0.02861057	0.53459119
MMP9	P14780	32	0.02343712	0.55555556
TLR4	O00206	31	0.0197468	0.56291391
ICAM1	P05362	30	0.00964587	0.5483871
REN	P00797	28	0.03262308	0.52469136
NOS3	P29474	26	0.02526339	0.52469136
MMP2	P08253	26	0.00836559	0.52795031
SMAD3	P84022	23	0.02238924	0.52469136
AGTR1	P30556	22	0.03230095	0.5
HMOX1	P09601	22	0.00833744	0.50898204
MUC1	P15941	20	0.03524761	0.49707602
ACE2	Q9BYF1	20	0.01064538	0.49418605
SNCA	P37840	19	0.05771326	0.49707602
B2M	P61769	19	0.02621018	0.51515152
MME	P08473	19	0.00918568	0.50295858
NFE2L2	Q16236	18	0.00676493	0.49418605
C3	P01024	16	0.02623523	0.46703297
RAC1	P63000	15	0.0264576	0.48571429
ZAP70	P43403	12	0.02368238	0.46448087
TRPV1	Q8NER1	11	0.01964922	0.45945946
HDAC4	P56524	9	0.05708607	0.47486034
BRD4	O60885	9	0.0114893	0.45212766

## Data Availability

The data used to support the findings of this study are included within the article.
